# ﻿An update to the 2014 nomenclator of Valvatidae

**DOI:** 10.3897/zookeys.1092.80548

**Published:** 2022-04-06

**Authors:** Gerhard Haszprunar

**Affiliations:** 1 SNSB – Zoologische Staatssammlung München, Münchhausenstr. 21, D-81247 München, Germany SNSB – Zoologische Staatssammlung München Munich Germany; 2 Faculty of Biology and GeoBio-Center of LMU Munich, Biozentrum, Großhaderner Str. 2, D-82152 Planegg-Martinsried, Germany Faculty of Biology and GeoBio-Center of LMU Munich, Biozentrum Munich Germany

**Keywords:** freshwater snails, Gastropoda, taxonomy, Valvatidae

## Abstract

This contribution is an update to the 2014 compilation of all supra- and (infra-) specific taxa of extant and fossil Valvatidae, a group of freshwater operculate snails near the base of Heterobranchia with a nearly worldwide distribution. This update includes corrections and many additions (two replacement taxon names, 21 mainly fossil taxa previously overlooked, and 37 invalid names) to the 2014 contribution and adds all newly described species (11) during the past eight years. The extensive reference list is directly linked, where possible, to the available electronic source of the cited papers.

## ﻿Introduction

My compilation of all taxa of extant and fossil valvatid gastropods (Haszprunar 2014) has been positively received by the scientific community and was followed by similar taxonomic work on other important groups of freshwater gastropods such as Neubauer (2016) for Melanopsidae or Neiber and Glaubrecht (2019) for Paludomidae. My work inspired and facilitated several recent papers on the taxonomy of Valvatidae. In particular, Russian authors cleared up the status of many uncertain taxa and provided valuable data and high-quality images of type material (e.g., Sitnikova et al. 2015, 2017; Vinarski 2016; Sitnikova 2018; Shirokaya et al. 2019; Andreeva et al. 2021; Osipova et al. 2021).

In contrast, the paleontological analysis of Valvatidae (and other taxa with similar shell morphologies) is still hampered by the unavailability of key literature. Many important and also quite recent taxonomic papers on fossil taxa have been written in non-Roman alphabets and often are not available as a digitized version. Thanks to the kindness of several colleagues, I could access some of the most important papers in particular by Russian or Chinese authorities, and the results of checking the original descriptions and their circumstances are compiled and reviewed herein.

Whereas there is constant progress in detecting and describing new valvatid species, concerning both fossil and extant taxa (Table 1), our understanding of species delimitation in Valvatidae generally is still limited. Also, a robust phylogeny of the family is still in its infancy, since only a few papers have provided molecular data on valvatids (e.g., Hauswald et al. 2008; Clewing et al. 2014; Saito et al. 2018; Falniowski et al. 2021). In particular, the seemingly well-known and widely distributed *Valvatapiscinalis* (O.F. Müller, 1774) is likely to represent a species complex spread all over the Palaearctic and (by introduction) also the Nearctic region.

**Table 1.** List of new names (alphabetically arranged) since publishing of the nomenclator (Haszprunar 2014).

(1) Newly described taxa:
Valvata (Tropidina) armeniaca Walther & Glöer, 2019
† *Valvataducati* Esu & Girotti, 2015
† *Valvatajiaolaiensis* Yu, Salvador, Wang, Fang, Neubauer, Li, Zhang, Wan, 2021
Valvata (Tropidina) kebapcii Odabaşi, Glöer & Yildirim, 2015
† *Valvatakoehleri* Harzhauser, Neubauer & Hosgör, 2018
*Valvatakournasi* Glöer & Hirschfelder, 2019
† *Provalvatamaior* Cataldo, Lazo, Luci & Aguirre-Urreta, 2019
† *Valvatamathiasi* Esu & Girotti, 2018
† *Provalvataminor* Cataldo, Lazo, Luci & Aguirre-Urreta, 2019
† *Valvatapyramidula* Esu & Girotti, 2015
† *Valvataheidemariaewillmanni* Neubauer, Harzhauser, Kroh, Georgopoulou & Mandic, 2014
(2) Previously overlooked taxa:
† *Valvataalta* K.A. Ali-Zade, 1932
† *Valvataandrussovi* A.A. Ali-Zade, 1967
† Valvata (Cincinna) arnaudi Repelin, 1902
† *Paludinaavia* Eichwald, 1853
† *Valvatabalchanica* A.A. Ali-Zade, 1967
Valvatapiscinalisvar.cancellata Baudon, 1884
Valvatabicarinatavar.connectans Walker, 1906
† *Valvatacosinensis* Stache, 1889
† *Valvataegregia* Noulet, 1857
† *Valvatafaujasii* Dumas, 1876
† Valvatavancianavar.kubanica Krestovnikov, 1931
† Valvata (Valvata) platispira Szőts, 1953
† *Valvatapolita* A.A. Ali-Zade, 1973
† Valvata (Cincinna) pontica Pană, 1990
† Valvata (Borysthenia) pronaticina Lindholm, 1932
† Valvata (Cincinna) rakovetzae Popova & Starobogatov, 1970
† Valvata (Cincinna) splendida Szőts, 1953
† *Valvatatanaiticus* Sanco, 2007
† *Valvataturbinata* Stache, 1889
† *Valvataturbinoides* K.A. Ali-Zade, 1936
† *Valvatauralica* Popov, 1965
(3) Other names that are not valid or available:
“*Valvatabaikalensis*” [*Valvatabaicalensis*]
“*Valvatacaliforniensis* Mss.” [nomen nudum]
“Cincinna (Sibirovalvata) chankensis Prozorova, 1988” [Cincinna (Sibirovalvata) hankensis]
† “*Valvataunicariniferachiknaformis*” [invalidly published]
† “Valvata (Cincinna) circinata (Greppin, 1855)” [*Paludinacircinata* Greppin, 1855; non *Valvatacircinata* Sandberger, 1871]
† “Valvatapiscinalisvar.cistopolitana G.I. Popov” [nomen nudum]
“*Valvatacupensis*” [*Valvatakupensis*]
† “*Valvatadensistriata*” [*Valvatadensestriata*]
“*Valvatabicarinatadepressa* Walker” [*Valvatabiarinataperdepressa* Walker, 1906]
“*Valvataeuzonia* Ziegler” [trade name]
† “Valvata (Cincinna) fuiensis” [†Valvata (Cincinna) fuxinensis Yü, 1987]
“*V.* [i.e., *Valvata*] *impressa*” [*Valvatadepressa*]
† “Valvata (Cincinna) joncheryacensis Wenz, 1923” [*Valvatajoncheryensis* Wenz, 1930]
“Valvata (Cincinna) aliena var. korotnewi Ldh. 1909” [*Valvatakorotnevi* Lindholm, 1909]
“*Valvatakurensis*” [*Valvatakupensis* Fuchs, 1870]
“*Valvatalanta*” [*Valvatalauta* Lindholm, 1909]
“*Valvataluguensis*” [nomen nudum]
† “Valvata (Cincinna) mengyinensis (Grabau)” [†*Bithyniamengyinensis* X *Valvatasuturensis*]
“*Valvatamontenegrinus* Glöer & Pešić, 2008” [*Valvatamontenegrina*]
† “*Valvatanikosi*” [*Pyrgulanikosi* Esu & Girotti, 2015]
† “*Valvatacristatapalustris* Kormos” [*Valvatacristata* X *Stagnicolapalustris*]
† “*Valvatacristatapslustris*” [*Stagnicolapalustris* (O.F. Müller, 1774)]
“*Costovalvatapulchra*” [nomen nudum]
“*Valvatapygrncea*” [*Valvatapygmaea*]
† “Valvata (Cincinna) rakovetzae” [† Valvata (Cincinna) racovetzae Popova & Starobogatov, 1970]
“*Valvataskniadica*” [†Valvata (Aphanotylus) skhiadica]
“*Valvataradiatulasubnaticina*” [*Valvatasubnaticina* Łomnicki, 1886]
“*Valvatacristata* monstr. *subscalaris*” [monstrosity]
“*Valvatatheotokii*” [*Valvatatheodokii*]
“*Liratinatongbinzhenensis*” [nomen nudum]
† “*Valvataturkmena*” [*Pyrgulaturkmena* Ali-Zade, 1967]
“Valvatapiscinalis(Müll.)var.uistopolitana Pop.” [*Valvatacistopolitana*]
† “*Planorbissymmetricus* Ludwig, 1865” [*Planorbissymmetrus* Ludwig, 1865]
† “*Valvataunicariniferaunicarinifera*” [invalidly published]
“*Valvatavaciani* Nourn.” [*Valvatavanciana* Tournouër, 1875]
“*Valvatavenciana*” [*Valvatavanciana* Tournouër, 1875]
“Cincinna (Cincinna) vinogradovkensis” [*Valvatavinogradokaensis* (Gozhik, 2002)]

As a result, there have not been any recent proposals of new genera or further revisions to the supraspecific classification. Accordingly, this update is limited to the species level. The present update adds two extant and nine fossil species recently described, two replacement taxon names, another 21 mainly fossil taxa previously overlooked, and nearly 40 names based on confusions, misspellings, or invalid publications (Table 1). In addition, numerous data on type localities or type material or sequence information are provided.

## ﻿General remarks

As in the previous work (Haszprunar 2014) all taxa (species, subspecies, named varieties) are listed alphabetically in their original version (only the spelling may be corrected according to the actual ICZN rules) regardless of validity, current taxonomic status, and synonymy. It is followed by either the citation to the page in my 2014 publication or indicated as a name newly treated herein. I also add available data sources (type material, anatomy, molecular data), which may be useful for future species delineation.

Two Ph.D. theses are discussed, since they contain the description of new taxa of Valvatidae (and of several other families): Bingle-Davis (2012) (University of North Dakota, U.S.A.) and Siodiropoúlou (2003) (Aristotle University of Thessaloniki, Greece). Both theses do not satisfy the criteria for publication of the International Code of Zoological Nomenclature, Articles 8.1 and 8.6. Students and faculty advisors should ensure that Ph.D. theses do not include proposed new taxa, except as, e.g., “Species A” so that these manuscript names will not enter the taxonomic literature and databases.

A widely cited Chinese-language work, “Youlou 1978” is particularly problematic. Previous authors, myself included, did not realize that this name actually means “Editors” or “Editorial Board” in Chinese, and no individuals are identified as the authors of either this publication or specific sections of this publication. I was uncertain about the true authorship of the very many taxon names of fossil gastropods introduced in this work. Indeed, the authorship “Youluo” appeared in all cases in the online type catalogue of the Nanjing Institute as well as in the Zoological Record, and has been repeatedly cited by later authors. Meanwhile I was able to check the original paper and can confirm the term “Youluo” as the true (i.e., printed in this way) authority in all cases [checked by a native Chinese colleague]. Most important, the actual names of any member of this group are not provided anywhere in this volume. However, ICZN Art. 14 clearly states that “A new name or nomenclatural act published after 1950 with anonymous authorship [Art. 50.1] is not thereby made available”. Accordingly, all species names (more than 120 new taxa!) as well as higher taxa (e.g., family Bohaispiridae and many hydrobiid genera; see Kabat and Hershler 1993) being introduced in this work are not available from there, but may have become available later by other authorities, if they clearly refer to the reference with an image of specimens or a diagnosis of the species. Among the Valvatidae they are listed in alphabetic order below and the names are marked as fossil taxa by “†”:

† Valvata (Cincinna) applanata Zhu X., 1995

† “*Liratinabasicarinata*”

† “*Tropidinabellireticulata*”

† “*Liratinafahaniuensis*”

† “*Liratinahedobia*”

† “*Aphanotylushumeratus*”

† “*Valvatamagniumbilicata*”

† “*Costovalvataminuta*”

† “Valvata (Atropidina) pileiformis”

† “*Liratinaqikouensis*”

† “Valvata (Cincinna) rehetaiensis” → *Valvatarehetaiensis* Zhu G.-X., 1980

† “*Valvataringentis*”

† “*Liratinatuozhuangensis*”

† “*Valvatazhouqingzhuangensis*”

Details on these names are outlined below under each name.

## ﻿Update of species names

The page number for the taxon name in my previous work (Nomenclator of Valvatidae: Haszprunar 2014) is given in parentheses as (Nom: ##).

***Valvataaliena*** C.A. Westerlund, 1877 (Nom: 16)

Remarks: Sitnikova et al. (2015: 3–4, fig. 1B) provided a photo of the lectotype and from specimens of several localities as well as an extensive and annotated citation record in the Russian literature. Andreeva et al. (2021: fig. 1A) added excellent photos from specimens of the Taz River basin (western Siberia).

***Valvataalpestris*** Küster, 1853 (Nom: 16)

Type locality: “in kleinen Seeen an der Quelle des Giessbaches ohnweit des Faulhorns bei Grindelwald” (Küster 1853: 87). According to current maps this is probably the “Schwarzseeli” near the Faulhorn at Grindelwald, Switzerland.

Types are figured by Boeters and Falkner (1998, not 2002 as stated).

† ***Valvataalta*** K.A. Ali-Zade, 1932 (NEW)

Original source: K.A. Ali-Zade 1932: 21, pl. 2: figs 12–14 (not seen, but according to Ali-Zade 1936: 17).

Type locality: near Naftalan, Azerbaijan.

Type horizon: Akchagylian, Upper Pliocene or Lower Pleistocene.

Remarks: Junior homonym of *Valvataalta* Deshayes, 1867, replaced by *Valvataturbinoides* K.A. Ali-Zade, 1936 (p. 17, pl. 1: figs 28–30).

† **Valvata (Cincinna) altaica** Popova & Starobogatov (in Popova, Devyatkin & Starobogatov, 1970) [not 1981 as stated in Haszprunar 2014] (Nom: 17)

Original source: Popova et al. 1970: 25, pl. 1: fig 2, pl. 2: figs 13, 14.

Type locality: Chuya Basin, left bank of the Chuya River, not located in Irkutsk Region as indicated in Haszprunar (2014), but in the southeastern part of Altai Mountains, Russia.

Type material: Holotype deposited in the Zoological Institute, of the Russian Academy of Sciences, St. Petersburg (No. ZIN 1/533-1968).

Remarks: Unfortunately this fossil species was omitted from the recent review of taxa created by Starobogatov (Sitnikova et al. 2017).

**Cincinna (Sibirovalvata) amurensis** Moskvicheva, 1985 (in Starobogatov & Zatravkin, 1985) (Nom: 17)

Type material: Holotype deposited in the Zoological Institute of the Russian Academy of Sciences, St. Petersburg (No. ZIN 1/405-1973).

Remarks: Saito et al. (2018) provided molecular data from specimens from Vladimiro-Petrovka (near southwest border of Khanka Lake), Primorsky region, Russia.

†***Valvataandreaei*** Menzel, 1904 (Nom: 18)

Type horizon: Quaternary.

Type material: not designated as such, but a sample with more than 20 specimens collected 1903 from the type localities (Alfeld an der Leine and Wallensen, Niedersachsen, Germany) with the original label stating “Zwergform von *piscinalis*” is stored in the Geozentrum Hannover (originally from Geowissenschaftliche Sammlungen Berlin, BGR) under BGR-B-STGR-000032180 (Alfeld) and BGR-B-STGR-000032693 (Wallensen).

“***Valvataandrezowski***” (GNI, GBIF) (Nom: 18)

Remarks: I reported about a label with this name in the Academy of Natural Sciences of Drexel University (Philadelphia). According to Bram van der Bijl (pers. comm. email 06 Feb 2014) the collection of the Biodiversity Center Naturalis (Leiden, Netherlands) holds a sample with a similar label.

† ***Valvataandrussovi*** A.A. Ali-Zade, 1967 (NEW)

Original source: A.A. Ali-Zade 1967: 224–225, pl. 84: figs 5, 6.

Type locality: Maly Balkhan, northwest Turkmenistan.

Type horizon: Upper Akchagylian, Lower Pleistocene.

Type material: Dr Pavel Frolov (pers. comm. via Dr. Paval Kijashko, 22 Feb 2022) said that the fossil molluscs described by A.A. Ali-Zade in 1967 are all stored in the Museum of Earth Sciences of Moscow State University (MES MSU).

Remarks: not treated in the last 50 years.

† ***Amplovalvatasuturalisanjipingensis*** Yü, 1980 (in Yü & Pan, 1980) (Nom: 18)

Original source: Yü and Pan 1980: 149, pl. 3: figs 1, 2.

† ***Amplovalvataantiqua*** Pan, 1980 (in Yü & Pan, 1980) (Nom: 19)

Original source: Yü and Pan 1980: 148, pl. 2: figs 21–24.

† **Valvata (Cincinna) applanata** Zhu X., 1995 (Nom: 20)

Original source: Zhu X. 1995: 79, pl. 22: 1–14, pl. 23: 9–12. Directly referred to Youluo 1978 (not available from there; see above).

Type locality: Qinghai Goulucuonan.

Type horizon: Cenozoic.

Type series: Nanjing Institute of Geology and Paleontology, Samples ## 118778-118786 and ## 118794-118797 (13 specimens).

***Valvataarenifera*** Lea, 1834 (Nom: 20)

Remarks: The history of misidentifications and nomenclature of this trichopteran insect with figures of the original description and with further links was presented by Lee (2015).

**Valvata (Tropidina) armeniaca** Walther & Glöer, 2019 (NEW)

Original source: Walther and Glöer 2019: 1–5: fig. 2 (1–3 shell of holotype, 9 and 10 shell of paratype), fig. 3 (environment of type locality).

Type locality: Armenia, Armavir Province, bridge over river Kasakh, 1.6 km W of Vagharshapat, 40.1650°N, 44.2558°E, 870 m asl, 27 Aug. 2018, F. Walther leg.

Type material: Holotype: Shell height 1.5 mm, width 2.5 mm, from type locality (Zoological Museum HamburgZMH 59491); Paratypes: from type locality (ZMH 59492, 3 specimens in ethanol); from type locality (F. Walther 13603; 1 dry shell); Ararat Province, Jrahovit, ditch S of the cemetery, 40.0450°N, 44.4880°E, 850 m asl., 26 Aug. 2018, leg. F. Walther (FW 14008, 1 specimen in ethanol).

† **Valvata (Cincinna) arnaudi** Repelin, 1902 (NEW)

Original source: Repelin 1902: 90, pl. 5: figs 40–42.

Type locality: Simeyroles, Département Dordogne in Nouvelle-Aquitaine, south France.

Type horizon: Cenomanian, Lowest Upper Cretaceous.

Type material: described from the collection Matheron, Museum nationale d´Histoire naturelle (MNHN), Paris.

† **Valvata (Cincinna) austrina** Pan, 1977 (Nom: 20)

Type locality: YH 5054-8-1, Mimalong, Lufeng County, Yunnan Province, China.

Type horizon: Fluvial-lacustrine horizon in the Zhanghe Formation, Bajocian, Jurassic.

Type material: Holotype at Nanjing Institute of Geology and Paleontology #24250.

† ***Paludinaavia*** Eichwald, 1851 (NEW)

Original source: Eichwald 1851: 136, pl. 10: fig. 28a–c; reprinted in Eichwald 1852: pl. 10: fig. 28a–c (atlas) and Eichwald 1853: 288 (text). Nosowska (2020: 455) recently outlined that the names of all illustrations of Eichwald (1852, 1853) had been made available in 1851 in a Russian version with valid descriptions and an identical atlas. Until this paper all authors did not realize that the 1851 publication was a book on its own due to the identical plate numbering (this book is nearly unknown in European libraries). The year 1859 on the cover of the atlas being digitized at the Biodiversity Heritage Library (see references) and cited by Janssen (1984) obviously concerns a later edition.

Type locality: near Kuncza (today Kuncha, region of Khmelnytskyi/ Chmelnyzkyj), Ukraine.

Type horizon: Neogene (details not provided).

Remarks: Frauenfeld (1864: 576) regarded this species as belonging to *Hydrobia*, but later it was considered to belong to *Amnicola*. Recently, however, it has been listed as “*Valvataavia* (Eichwald, 1853)” by Neubauer et al. (2014c: Supplement 1). “Paludinacf.avia Eichw.” was found by Stiny (1924) in Tertiary layers at several locations near Feldbach in Styria (Austria).

***Valvatabaicalensis*** Gerstfeldt, 1859 (Nom: 21)

Remarks: Sitnikova (1991: 64–65, fig. 4) described and figured the egg capsules. Saito et al. (2018) provided molecular data from specimens from the type locality, Lake Baikal (Listvyanka), Russia. Sitnikova (2018: fig. 6A) illustrated the lectotype.

“***Valvatabaikalensis***” mentioned in Bogachev (1961: 152) (NEW)

Remarks: misspelling of *Valvatabaicalensis* Gerstfeldt, 1859.

† ***Valvatabalchanica*** A.A. Ali-Zade, 1967 (NEW)

Original source: A.A. Ali-Zade 1967: 224, pl. 84: figs 1–4.

Type locality: Maly Balkhan, northwest Turkmenistan.

Type horizon: Akchagylian, Upper Pliocene to Lower Pleistocene.

Type material: Dr Pavel Frolov (pers. comm. via Dr. Paval Kijashko, 22 Feb 2022) stated that the fossil molluscs described by A.A. Ali-Zade in 1967 were all stored in the Museum of Earth Sciences of Moscow State University (MES MSU).

Remarks: not treated in the last 50 years.

† “***Liratinabasicarinata***” mentioned in Youluo 1978 (Nom: 22)

Remarks: Not available from there (see above). I could not find a subsequent full reference on this species.

***Valvatabathybia*** W. Dybowski, 1886 (Nom: 22)

Remarks: Type material unknown. Sitnikova (2018: fig. 2B) figured a topotype and further specimens from various localities from Lake Baikal.

† “***Tropidina ? bellireticulata***” mentioned in Youluo (1978) (Nom: 23)

Remarks: Not available from there (see above) or from Ye et al. (1996: 37; nomen nudum, no reference). I could not find a subsequent full reference on this species that would validate the name.

***Valvatabeltrani*** Contreras-Arquieta, 1993 (Nom: 23)

Remarks: Contreras-Balderas and Lozano-Vilano (1996) reported that this species, which lived in isolated springs in North Mexico, had become extinct at the time of discovery by the drying of the springs due to agricultural needs.

† ***Valvatabeysehirensis*** Glöer & Girod, 2013 (Nom: 23)

Type material: Holotype deposited in Zoological Museum Hamburg (ZMH 79381). Paratypes: 3 shells in Museo Civico di Storia Naturale, Milano, Italy (MSNM Mo-36591), numerous shells in the collection of Alberto Girod (AGMal 3595), 3 shells in collection Glöer (Hettlingen, Germany).

† “***Valvataheidemariaebicarinata*** Willmann, 1981” (Nom: 24)

Type material: According to Neubauer et al. (2014b: 22) deposited in the Geological-Paleontological Institute, University of Kiel, no number indicated.

Remarks: As outlined, the name is a junior homonym of *Valvatabicarinata* Lea 1841. Accordingly, the species has been renamed as *Valvataheidemariaewillmanni* Neubauer, Harzhauser, Kroh, Georgopoulou & Mandic, 2014.

**Valvata (Cincinna) biwaensis** Preston, 1916 (Nom: 25)

Remarks: Saito et al. (2018) provided molecular data from specimens from the type locality, Lake Biwa, Japan.

**Valvata (Cincinna) brandti** Westerlund, 1897 (Nom: 26)

Type material: Walther and Glöer (2019: 4) checked the taxonomy of the species and although they did not have contact to type material, they concluded that “The original description of *V.brandti* is based on two lots. One was collected by A. Brandt in Lake Sevan, while the other was found by L. Młokosiewicz near Lagodekhi in Georgia. Between both localities is a distance of more than 150 km. The Lagodekhi record belongs to *Caspicyclotussieversi* .... (Cyclophoridae)…..The other lot, however, seems to belong to *Valvatapiscinalis*, which is known to occur in Lake Sevan (e.g., Mashkova et al. 2018)”.

Dr Pavel Kaijashko (pers. comm. 22 Feb 2022) provided the following additional valuable information: “Indeed, the original description of *V.brandti* is based on two lots … [mentioned by Walther and Glöer (2019)]. In 1912 W. Lindholm [Lindholm 1912] redefined the Lagodekhi finds and placed them to *Cyclotussieversi* (now *Caspicyclotussieversi*). The other lot he attributed to the genus *Valvata*. There is a handwritten entry by W. Lindholm in the ZIN RAS catalogue about this. The specimens of *V.brandti* collected by A. Brandt are poorly preserved. Nevertheless, their conchological features (shell size, shape and sculpture of the whorls, diameter of the umbilicus) indicate belonging to Valvata (Tropidina), but not to *V.piscinalis*.”

**Valvata (Cincinna) aliena var. brevicula** Kozhov, 1936 (Nom: 26)

Type material: Sitnikova et al. (2004) designated a lectotype, which was later illustrated by Sitnikova et al. (2015: fig. 1D). However, Vinarski and Kantor (2016: 274) reported that only syntypes but no lectotype could be found at the Zoological Institute, Academy of Sciences, St. Petersburg (ZIN). Thus, it remains unclear whether or not the designated lectotype has been lost.

Remarks: Clewing et al. (2014, supplementary material) provided molecular data (as RU05/1). Sitnikova et al. (2015: 13–15) provided photographs of specimens from several localities as well as an extensive and annotated citation record in particular of the Russian literature. Recently, Andreeva et al. (2021: fig. 3B) added excellent photographs from specimens of the Taz River basin (western Siberia).

“***Valvatacaliforniensis*** Mss.” [manuscript] mentioned in Schmeltz (1869, IV: 75) (NEW)

Remarks: A nomen nudum like many other similar cases in the catalogue of the Godeffroy Museum (Bieler and Petit 2012: 46; #5353). Interestingly, a specimen with this name and “from California” was offered ten years later for 30 Pfennig in the “Tausch-Catalog” of the Nachrichtsblatt der Deutschen Malakozoologischen Gesellschaft (1879, Vol. 11: 102).

***Valvatapiscinalisvar.cancellata*** Baudon, 1884 (NEW)

Original source: Baudon 1884: 293, pl. 10: fig. 5.

Type locality: Département de l Oise, France.

† ***Valvatacangshanensis*** Pan, 1982 (Nom: 28)

Original source: Pan 1982: 430–431, pl. 1: figs 24–27.

† “***Valvatacarinata*** Fuchs, 1870” (Nom: 28–29)

Remarks: As stated in Haszprunar (2014), a junior homonym of *Valvatacarinata* Sowerby, 1834. Accordingly, the name has been meanwhile replaced by Neubauer et al. (2014a) by *Muellerpaliapseudovalvatoides* Neubauer, Harzhauser, Kroh, Georgopoulou & Mandic, 2014 (Hydrobiidae).

“**Cincinna (Sibirovalvata) chankensis** Prozorova, 1988” (NEW)

mentioned in Prozorova 1992: 106–107 (fig. 8 legend).

Remarks: Misspelling of Cincinna (Sibirovalvata) hankensis Prozorova, 1988.

***Cincinnachersonica*** Chernogorenko & Starobogatov, 1987 (Nom: 29)

Type material: Vinarski and Kantor (2016: 270) stated that the “holotype” in the Zoological Institute of the Russian Academy of Sciences, St. Petersburg (ZIN 1/41-1974) mentioned by Kantor et al. (2011: 66) is in fact a paratype and that the holotype is missing.

† “***Valvataunicariniferachiknaformis***” (NEW)

mentioned in Bingle-Davis (2012: 104)

Horizon: Upper Cretaceous.

Locality: near the villages of Butera and Machhaghoda (Chhindwara District: Madhya Pradesh) ca. 160 km north of Nagpur (22.11 N, 79.14 E), eastern Dekkan Plateau, India.

Material: “Holotype”: InS1199 (Appendix 2 (Nom: 137), fig. N; 6.8 mm × 7.6 mm), deposited at University of North Dakota, Grand Forks, North Dakota, USA.

Remarks: This subspecies name was mentioned in an unpublished PhD thesis (no ISBN or ISSN numbering) and thus is not formally described as required by ICZN Art. 8.1.3 and 8.6.

† “**Valvata (Cincinna)**” ***circinata*** (Greppin, 1855) mentioned in Wenz (1928: 2427) (NEW / Nom. 30)

Original source: Greppin 1855: 29, 71, pl. 3: fig. 11a–c (as *Paludinacircinata*)

Type locality: Limestone banks von Sornetan and Tramelan, Val de Délemont/Bezirk Delsberg, Kanton Jura, Switzerland.

Type horizon: Lower to middle Miocene.

Remarks: Referred to Merian (1849: 34, mentioned there as *Paludinacircinata*, a nomen nudum). ICZN Art. 50.1.1. states clearly that “If the identity of that other person is not explicit in the work itself, then the author is deemed to be the person who publishes the work”, making Greppin the author of *Paludinacircinata*. However, contrary to the classification by Wenz (1928: 2427) this taxon with an extended large whorl and a broad apertural lip is certainly not a valvatid, but much more likely a *Lithoglyphus*. It is also not identical to those specimens from a nearby locality, which are illustrated and described by Sandberger (1870–1875: 324, pl. 18: fig. 5a–c) as *Valvatacircinata* (and also referred to Merian). Accordingly, both *Paludinacircinata* Greppin, 1855 and *Valvatacircinata* Sandberger, 1871 remain valid taxa.

† “***Valvatapiscinalisvar.cistopolitana*** G.I. Popov” (NEW)

cited as “*cistopolitana* nov.”, a nomen nudum in Goretsky (1964: 55) and secondarily by Balabanov et al. (2010: 196).

Remarks: I could not find any trace of this name in the papers of G.I. Popov or in subsequent papers, accordingly a “taxon inquirendum”, probably a nomen nudum, not available.

**Valvata (Cincinna) confusa** (Westerlund, 1879) (Nom: 31)

Remarks: Clewing et al. (2014: supplementary material) provided molecular data (as RU08/1). Saito et al. (2018) provided additional molecular data from specimens from Delga River, Khuvsgul, Mongolia. Andreeva et al. (2021: fig. 1B) added excellent photographs from specimens of the Taz River basin (western Siberia).

***Valvatabicarinatavar.connectans*** B. Walker, 1906 (NEW)

Original source: Walker 1906: 30 (not figured, described as unicarinate).

Type location: Lake Michigan, New Buffalo, Michigan, USA.

Type material: No. 24142 of coll. Walker (presumably now in University of Michigan Museum of Zoology). Alan Kabat (pers. comm. 30 Jan 2022) could not find it in the UMMZ database, but the sample may not yet be catalogued online.

***Neritacontorta*** Müller, 1774 (Nom: 32)

Type material: Nekhaev et al. (2015) and Vinarski and Kantor (2016: 271) stated that they could not find type material in the Zoological Museum of Copenhagen, where Kantor et al. (2011: 66) had suggested it might exist.

† ***Valvatacosinensis*** Stache, 1889 (NEW)

Original source: Stache 1889: 117, pl. 2: fig. 26.

Type locality: In old coalmines, north of Cosina (today Hrpelje-Kozina) [5 km east of Trieste, Italy], Slovenia.

Type horizon: *Stomatopsis* horizon, Paleocene (see Jurkovšek et al. 2016: 358).

Remarks: Stache himself noted the high similarity with *Valvatapupoidea* Gould, 1961, currently considered as *Lyogyruspupoideus* (Gould, 1841) (Amnicolidae).

“***Valvatacupensis***” mentioned in Bogachev (1961: 73) (NEW)

Remarks: misspelling of *Valvatakupensis* Fuchs, 1870.

† ***Aphanotylusdakangensis*** Pan, 1982 (Nom: 35)

Original source: Pan 1982: 432, pl. 2: figs 5–8.

† ***Valvata? dalaziensis*** Zhu G.-X., 1980 (Nom: 35)

Original source: Zhu 1980: 38, pl. 14: figs 24–26.

Type locality: Yanji, Jilin, Northeast [not northwest as stated in Haszprunar 2014] China.

Type horizon: Dalazi Formation, Lower Cretaceous.

† ***Valvatadecollata*** Hislop, 1859 (Nom: 36)

Type horizon: Upper Cretaceous (not Tertiary as stated by Hislop).

Type material: Lectotype designated and figured by Hartman et al. (2008: fig. 18A–C), reprinted by Bingle-Davis (2012: 132), deposited under PIMG 1188 (Palaeo Invertebrate Mesozoic Gastropod) at the Natural History Museum of the United Kingdom (NHMUK), the original Latin description was translated to English.

† ***Amplovalvatadeformis*** Pan, 1980 (in Yu & Pan, 1980) (Nom: 36)

Original source: Yu and Pan 1980: 149, pl. 2: figs 25, 26.

† ***Valvata*** (***Cincinna***) ***delaunayi*** Cossmann, 1907 (Nom: 36)

Type material: Museum National d'Histoire Naturelle , Paris (MNHN F-J08320 (holotype) and F-J08321 (paratype).

***Valvata (Liratina) baicalensis var. demersa*** Lindholm, 1909 (Nom: 37)

Remarks: Sitnikova (1991: 64, fig. 3) described and figured the egg capsules. Sitnikova (2018: fig. 6G) illustrated the lectotype and specimens from various localities in Lake Baikal (fig. 8A–F, H).

† “***Valvatadensistriata***” mentioned in Henderson (1935: 296) (NEW)

Remarks: Misspelling of *Valvatadensestriata* Pilsbry, 1934 (Nom: 37).

“***Valvatabicarinatadepressa*** Walker” mentioned by Sterki (1907: 387) (NEW)

Remarks: Misspelling of *Valvatabicarinataperdepressa* Walker, 1906.

† ***Valvata*** (***Tropidina***) ***donghucensis*** Pan, 1977 (Nom: 38)

Type locality: YH 5033, Donghucun, Lufeng County, Yunnan, China.

Type horizon: Fengjiahe Formation, Jurassic (201.6 to 175.6 Mya).

† ***Valvatadromica*** Fontannes, 1881 (Nom. 39)

Remarks: Currently considered as *Pseudamnicoladromica* (Fontannes, 1881) (Hydrobiidae) (Neubauer et al. 2014c).

† ***Valvataducati*** Esu & Girotti, 2015 (NEW)

Original source: Esu and Girotti 2015b: 151–152, figs 3–5. Previously mentioned and illustrated as “*Valvata* sp. nov.” by Ciangherotti et al. (1997: 307, pl. 1: fig. 3).

Type locality: Stirone River section, between Laurano and San Nicomede, Emilia, northern Italy.

Type horizon: Lower Middle Pleistocene.

Type material: stored in Senckenberg Museum Frankfurt (Holotype SMF 345836, paratypes SMF 345837/2).

† ***Valvataegregia*** Noulet, 1857 (NEW)

Original source: Noulet 1857: 12 (no figure).

Type locality: Calcaire de Villeneuve-la-Comtal et du Mas-Saintes-Puelles (Département Aude), southwest France.

Type horizon: Upper Eocene.

Remarks: Currently considered as *Physotremaegregia* (Noulet, 1857), a terrestrial species of the architaenioglossan family Craspedopomatidae.

“***Valvataeuzonia*** Ziegler” mentioned in Baudon (1884: 293) (NEW)

Remarks: one of the many unavailable names created by the Viennese shell dealer Franz Andreas Ziegler (see Rossmässler 1837: 32, legend to pl. 26: fig. 356, footnote; Schmidt 1846).

† ***Liratinafahaniuensis*** Zhu G.-X., 1980 [non Youluo 1978] (Nom: 41)

Original source: Zhu 1980: 39, pl. 19: fig. 4, referred to Youluo (1978).

Remarks: Not available from Youluo 1978 (see above), but Zhu (1980) fulfils all requirements of validation (description and figure).

Type locality: Xinmin, northeast China.

Type horizon: The lower part of the first section of the Eocene–Oligocene Shahejie Formation.

***Cincinnafalsifluviatilis*** Starobogatov in Anistratenko V.V. and Anistratenko O.Yu., 2001 (Nom: 41)

Original source: Anistratenko V.V. and Anistratenko O.Yu. 2001: 139–140, fig. 110 (as *Cincinnafalsifluviatilis*; description by Starobogatov pro *Valvatafluviatilis* sensu Westerlund, 1886: 34 (actually, 134); non Colbeau, 1859) [see Sitnikova et al. (2017: 260)].

Type locality: The locality of the lectotype is unfortunately not provided by Vinarski and Kantor (2016: 268).

Type material: Lectotype in the Zoological Institute of the Russian Academy of Sciences, St. Petersburg (ZIN), #1 in the systematic catalogue under the name.

Remarks: Originally the name was erected to replace “*Valvatafluviatilis* sensu Westerlund, 1886”. However, as outlined by Haszprunar (2014) a “sensu” name is not available, thus cannot be replaced. On the other hand, Vinarski and Kantor (2016: 268) recently found the original material of Westerlund mentioned by Anistratenko and Anistratenko (2001) and designated a lectotype. Accordingly, the requirements of ICZN Art. 16.4 are fulfilled to name this species.

† ***Valvatafaujasii*** Dumas, 1876 (NEW)

Original source: Dumas 1876: 462, referred to: “Mém. De Faujas, etc. …., t. xiv, pl. 19: figs 13–17” (i.e., Faujas de Saint Fond 1809).

Type locality: coal mine near Saint-Paulet (de-Caisson), Départment du Gard, south France.

Type horizon: Paulétien, Upper Cretaceous.

Remarks: Faujas de Saint Fond (1809) did not provide a name. According to Repelin (1902: 89, as *V.faujasi*) the taxon is a junior synonym of *Valvataminuta* Draparnaud, 1805, currently considered as *Islamiaminuta* (Draparnaud, 1805) (Hydrobiidae).

***Valvata*** (***Tropidina***) ***fezi*** Altimira, 1960 (Nom: 42)

Remarks: Arconada and Ramos (2002) designated this hydrobiid species as the type species of their newly erected genus *Spathogyna* and provided SEM photographs of the shell, protoconch, radula, and body surface as well as anatomical data.

***Valvatafrigida*** Westerlund, 1873 (Nom: 43)

Remarks: Andreeva et al. (2021: fig. 5B–D) added excellent photos from specimens of the Ratta river and Taz River basin (western Siberia).

† “***Valvata*** (***Cincinna***) ***fuiensis***” mentioned in Wan et al. (2013: 464) (NEW)

Remarks: Misspelling of Valvata (Cincinna) fuxinensis.

***Valvata (Cincinna) gafurovi*** Izzatullaev, 1977 (Nom: 44)

Type material: Holotype and 1 paratype in the Zoological Institute of the Russian Academy of Sciences, St. Petersburg (ZIN), ##1, 2 in the systematic catalogue under the name. Nineteen further paratypes are listed by Vinarski and Kantor (2016: 271). Holotype figured by Shirokaya et al. (2019: fig. 13C).

Remarks: Sitnikova (1983) described in detail the reproductive system and placed the species in the subgenusPamirocincinna Sitnikova & Starobogatov in Sitnikova, 1983. Shirokaya et al. (2019: 238) followed this replacement and provided a detailed bibliography of the species.

† “***Valvataantiquavar.gigas***” mentioned by Goretsky (1956a: 33) (Nom: 45)

Remarks: mentioned as “sp. nov.”, but is a nomen nudum like several other species names in the same paper. Also mentioned some years later by Bogachev (1961: 85, 91, 93: *Valvataantiqua* - morpha *gigas*), again without any description or image.

“***Valvata*** (***Jekeliusiana***) ***oecsensishalavatsi*** Gozhik, 2002” (Nom: 49)

Type locality: in Ukraine.

Remarks: Neubauer et al. (2014b: 20) pointed out that the original name “*öcsensis*” (Soós, 1934: 189) was erroneously emended to “*oecsensis*”. However, the correct emendation following ICZN rules is “*ocsensis*”, since it is not derived from a German but a Hungarian expression (ICZN 32.5.2.1). Current status: Valvata (Jekeliusiana) ocsensis halavatsi Gozhik, 2002.

***Cincinna*** (***Sibirovalvata***) ***hankensis*** Prozorova, 1988 (Nom: 49)

Original source: Prozorova 1988: 1936–1938, figs 1 (shell), 2 (spawn).

Type material: Holotype (ZIN 1/514-1986) and 6 paratypes (ZIN 2/514-1986) are deposited in the Zoological Institute of the Russian Academy of Sciences, St. Petersburg (ZIN).

† “***Liratinahedobia***” mentioned in Youluo 1978 (Nom: 50)

Remarks: Not available from there (see above) or from Ye et al. (1996: 48; misspelled as Liratina?helobia; nomen nudum, no reference). I could not find a subsequent full reference on this species that would validate the name.

***Valvatalewisivar.helicoidea*** Dall, 1905 (Nom: 50)

Remarks: Andreeva et al. (2021: 13, figs 1C, 2) confirmed the presence of this species in Siberia and added excellent photographs from specimens of the Taz River basin (western Siberia).

***Valvatacristatahokkaidoensis*** Miyadi, 1935 (Nom: 52)

Remarks: Saito et al. (2018) provided molecular data from specimens from the type locality, Lake Toro, Hokkaido Prefecture, and from Doba River, Aomori Prefecture, Japan. These data suggest that this species name actually encompasses multiple species. A short live movie of this species by Akira Ooyagi is presented at www.youtube.com/watch?v=f6p3w4WWgG4.

† ***Valvatahomalogyra*** Brusina, 1874 (Nom: 52)

Type material: According to Neubauer et al. (2016: 18, fig. 2A–C) “The syntype series includes 14 specimens [Croatian Natural History Museum in Zagreb] (NHMZ #1613 and NHMZ #1625.1–13) from Goručica SW Sinj (= Ruduša) described by Brusina (1874) but not illustrated due to bad preservation. Brusina (1897) subsequently illustrated only one specimen from Miočič. This misled Milan et al. (1974) to regard Miočič as the type locality and the specimen, which was available for the present study, as the “holotype” [fig. 2A–C]. Yet, surprisingly, the latter is apparently not even the specimen documented by Brusina (1897)”.

Remarks: Brusina (1874: 90) himself regarded *Valvatahomalogyra* as closely related to the extant *Valvataerythropomatia* Hauffen, 1856. As outlined (Haszprunar 2014: 39), the latter species became type of *Erythropomatiana* Radoman, 1978, a genus name currently considered as a junior synonym of *Hauffenia* Pollonera, 1898 (Hydrobiidae). Accordingly, it is also likely that *Valvatahomalogyra* is in fact a hydrobiid.

† “***Aphanotylushumeratus***” mentioned in Youluo 1978 (Nom: 53)

Remarks: Not available from there (see above) or from Ye et al. (1996: 49; nomen nudum, no reference). I could not find a subsequent full reference on this species that would validate the name.

“***V.* [i.e., *Valvata*] *impressa***” (NEW) Mentioned by Bogachev (1961: 91) and as “*V.impressa* Pfeff.” [Pfeffer?] by Goretsky (1964: 55) in the chapter Конхилиофауна кинельских отложений [Conchiliofauna of the Kinel-Lagerstätten] available at http://www.bibliotekar.ru/5-prareki-chetvertichnyi-period/7.htm

Remarks: I could not find any description of a “*Valvataimpressa*”. The malacologist Georg Pfeffer (1854–1931) did not describe any *Valvata* species. The name is likely a double misspelling of *Valvatadepressa* Pfeiffer, 1821.

***Valvatainconspicua*** C.B. Adams, 1851 (Nom. 55)

Remarks: currently considered as *Nanivitreainconspicua* (C.B. Adams, 1851) (Cochliopidae) (Jaume and Abbott 1948).

† ***Borystheniaintermedia*** Konrashov, 2007 (Nom: 56)

Type material: Holotype deposited at Paleontological Institute, Russian Academy of Sciences (PIN) #5148/1.

† ***Borystheniajalpuchense*** Gozhik, 2002 (Nom: 57)

Type locality: near Vinogradovka village, Odesa oblast, Bolhrads’kyi district, Ukraine.

Type horizon: Miocene, Middle Pontian.

Type material: Holotype (coll. Gozhik, #3162) figured by Osipova et al. (2021: fig. 3H).

Remarks: The species name should be *jalpuchensis*, since *Borysthenia* is feminine. Morphometric data of the shell compared with *Borystheniamenkeana* (Jelski, 1863) were provided by Osipova et al. (2021).

***Valvatajaponica*** Martens, 1877 (Nom: 57)

Type material: 1 syntype (of 2) from the Museum für Naturkunde, Berlin (ZSM # 38883 from the type locality, i.e., Hakone Lake) is figured by Vinarski (2016: 7, fig. 4E, F).

Remarks: Saito et al. (2018) provided molecular data of specimens from Doba River (Amori Prefecture) and Sagami-gawa River (Nagano Prefecture), Japan. These data suggest that this species name actually encompasses several biological species.

† ***Valvatajiaolaiensis*** Yu, Salvador, Wang, Fang, Neubauer, Li, Zhang & Wan, 2021 (NEW)

Original source: Yu et al. 2021: 5, fig. 3M–P.

Type locality. LK-1 borehole (36°15'55"N, 119°57'04"E), northern part of Jiaozhou City, Shandong Province, China.

Type horizon: Uppermost Cretaceous, Jiaozhou Formation; sample taken at a depth of 370.5 m.

Type material deposited in Nanjing Institute of Geology & Palaeontology: Holotype: NIGP #168642, paratype: NIGP #16864.

† ***Amplovalvatajingguensis*** Pan, 1977 (Nom: 57)

Original source: Pan 1977: 118, pl. 5: fig. 18.

Type locality: YHS492, Heping township, Jinggu County, Yunnan Province, China.

Type horizon: Bajocian/Bathonian fluvial-lacustrine horizon in the Hepingxiang Formation of China, Middle Jurassic (171.6–164.7 Mya).

Type material: Holotype deposited at Nanjing Institute of Geology & Paleontology NIGP #24247.

† “**Valvata (Cincinna) joncheryacensis** Wenz, 1923” (NEW)

Mentioned in Le Renard and Pacaud (1995: 98) and in Worldwide Mollusc Species Data Base (WMSDB).

Remarks: Misspelling of †*Valvatajoncheryensis* Wenz, 1930. Wenz (1923) only included pulmonate species.

† ***Aphanotylusjurassicus*** Pan, 1980 (in Yü & Pan, 1980) (Nom: 58)

Original source: Yü and Pan 1980: 150, pl. 3: figs 15, 16.

***Cincinnakamchatica*** Prozorova & Starobogatov, 1998 (Nom. 58)

Type material: Holotype (ZIN 1/97-1911) and 21 paratypes (ZIN 2/97-911) in Zoological Institute of the Russian Academy of Sciences, St. Petersburg (ZIN).

† **Valvata (Mesovalvata) karameilica** Wei, 1984 (Nom: 58)

Original source: Wei in Xinjiang Dizhi Ju 1984, 84, pl. 49: figs 1, 2.

Type locality: Yandi’s booth: Qitai Jubei Yingxun, Xinjang province, China.

Type horizon: Quishan Street, Upper Triassic.

**Valvata (Tropidina) kebapcii** Odabasi, Glöer & Yildirim, 2015 (NEW)

Original source: Odabaşi et al. 2015: 137, figs 2–4, 11 (shell), 5 (head), 6 (operculum).

Type locality: Turkey, northwestern Anatolia, Ayvacik town, Tuzla Stream, 39°31'30.8264"N, 26°17'9.57"E, 81 m altitude.

Type material: Holotype COMULM-G 0050 (also figured by Walther and Glöer 2019: fig. 2.7, 2.8 and by Glöer 2019: 210, fig. 264), 5 paratypes COMULM-G 0051, Çanakkale Onsekiz Mart University, Turkey (COMULM).

***Cincinnakizakikoensis*** Fujita & Habe, 1991 (Nom: 59)

Remarks: Saito et al. (2018) provided molecular data of specimens from Lake Nakatsuna, Nagano Prefecture, Japan.

**Valvata (Costovalvata) klemmi** Schütt, 1962 (Nom: 59).

Type material: A paratype (FS/8894) is stored in the Biologiezentrum Linz (Austria) (Aescht 2003).

**Valvatafluviatilisvar.kliniensis** Milachevich/Milaschewitsch, 1881 (Nom: 59)

Type locality: According to Vinarski and Kantor (2016: 267), the type locality “Moujevo” near Moscow is currently named “Muzhevo” and is at approx. 56°27´14"N, 36°50´54"E.

Type material: 56 syntypes in the Zoological Institute of the Russian Academy of Sciences, St. Petersburg (ZIN), #1 in systematic catalogue.

**Cincinna (Sibirovalvata) klucharevae** Starobogatov, 1985 (Nom: 60)

Type material: There are no type specimens in the collection of the Zoological Institute of the Russian Academy of Sciences (ZIN). The specimen with number ZIN 1/523-2014 is a topotype that is not included in the type series.

† ***Valvatakoehleri*** Harzhauser, Neubauer & Hoşgör, 2018 (NEW)

Original source: Harzhauser et al. 2018: 362–364, fig. 4 (shell and protoconch SEM).

Type locality: Kömürlü (40°46'14.74"N, 42°18'21.05"E, WGS84), Oltu-Narman Basin, northeastern Turkey.

Type horizon: Marly silt and sand of the upper Susuz Formation; Upper Oligocene or Lower Miocene.

Type material: Holotype (NHMW 2018/0019/0015) and paratypes (NHMW 2018/

0019/0016-0019), all from type locality and horizon, are deposited at the Naturhistorisches Museum, Wien (NHMW).

**Valvata (Cincinna) korotnevi** Lindholm, 1909 (Nom: 60)

Remarks: Remarks: Sitnikova et al. (2015: 3–9, fig. 1C) provided photographs of the lectotype and of specimens from several localities as well as an extensive and annotated citation record of the Russian literature in particular. Recently, Andreeva et al. (2021: fig. 3A) added excellent photographs from specimens of the Taz River basin (western Siberia).

“**Valvata (Cincinna) aliena var. korotnewi** Ldh. 1909” mentioned in Kozhov (1936: 17, 18). (NEW)

Remarks: Misspelling of Valvata (Cincinna) korotnevi Lindholm, 1909, correctly listed by Sitnikova et al. (2015: 11).

***Valvatakournasi*** Glöer & Hirschfelder, 2019 (NEW)

Original source: Glöer and Hirschfelder 2019: 18–21, figs 40–42 (shell), fig. 43 (maps), figs 44–46 (type locality).

Type locality: Nómos Chaniá, outflow of Lake Kournas, 3 km southeast of Georgioúpoli, Crete, Greece.

Type material: Holotype (ZMH 140040: also figured by Glöer 2019: 211, fig. 265) and 1 paratype (ZMH 140041) are stored in the Zoological Museum of Hamburg, Germany (ZMH), remaining paratypes (9 + 1 juv.) in coll. Hirschfelder (Kelheim, Germany).

Remarks: The anatomy of this species is unknown because only subrecent (fossil or dead) shells have been found.

***Megalovalvatakozhovi*** Sitnikova, 1983 (Nom: 60)

Remarks: Sitnikova (2018) illustrated the holotype (fig. 6H) and paratypes (fig. 10G–J) from various localities of Lake Baikal.

† **Valvatavancianavar.kubanica** Krestovnikov, 1931 (NEW)

Original source: Krestovnikov 1931: 20, pl. 2: figs 19–25. Redescribed and figured by Yakhimovich et al. (2000: 65, pl. 4: figs 7–10) as *Valvatakubanica* Krestovnikov, 1929

Type locality: Sediments of Estuary of Velykyi Kuyalnik River near Odessa, northwest Black Sea, Ukraine.

Type horizon: Pleistocene.

“***Valvatakurensis***” mentioned in Bogachev (1961: 73) (NEW)

Remarks: Misspelling of *Valvatakupensis* Fuchs, 1870.

**Valvata (Pseudomegalovalvata) laethmophila** Bekman & Starobogatov, 1975 (Nom: 61)

Remarks: Sitnikova (2018: fig. 2D) figured the holotype.

“***Valvatalanta***” mentioned in Bogachev (1961: 152) (NEW)

Remarks: Misspelling of *Valvatalauta* Lindholm, 1909.

† **Valvata (Cincinna) andreana var. latior** Menzel, 1904 (Nom: 62)

Type horizon: Quaternary.

Type material (more than 20 specimens without designation as types) is stored in the Geozentrum Hannover (originally from Geowissenschaftliche Sammlungen Berlin, BGR) starting with # BGR-B-ORIG-000181564 to # BGR-B-ORIG-000181888.

***Valvatalietuvensis*** Chernogorenko & Starobogatov, 1987 (Nom: 64)

Type material: Holotype (ZIN 1/601-1986) and 10 paratypes (ZIN 2/601-1986 and ZIN 3/601-1986) in the Zoological Institute of the Russian Academy of Sciences, St. Petersburg (ZIN). The type series includes the holotype and 10 paratypes. Holotype figured by Sitnikova et al. (2017: 257, fig. 3A–C). Additional data by Vinarski and Kantor (2016: 264).

“***Valvataluguensis***” mentioned by Du et al. (2017: 871) (NEW)

Locality: Lake Lugu (alpine, 2690 m above sea level), with the middle of the lake forming the border between the Ninglang County of Yunnan Province and the Yanyuan County of Sichuan Province, China.

Remarks: Not described or figured by the authors or subsequently, thus a nomen nudum. According to Wiese et al. (2020: 1101) this species might be endemic to Lake Lugu. Clewing et al. (2014) have previously provided molecular data of this lineage as “*Valvata* sp. from Lake Lugu”, resp. “clade CN07/1”. According to their analysis the species belongs to the subgenus Tropidina.

† ***Amplovalvatamagna*** Pan, 1980 (in Yu & Pan, 1980) (Nom: 65)

Original source: Yu and Pan 1980: 150, pl. 2: figs 5, 6.

† “***Valvatamagniumbilicata***” mentioned in Youluo 1978 (Nom: 65)

Remarks: Not available from there (see above). I could not find a subsequent reference on this species that would validate the name.

† ***Provalvatamaior*** Cataldo, Lazo, Luci & Aguirre-Urreta, 2019 (NEW)

Original source: Cataldo et al. 2019: 453–455, figs 6 (9–12), 7 (9–12).

Type locality: Quebrada del Gastrópodo, Mendoza Province, Argentina.

Type horizon: La Tosca Member, Huitrín Formation, facies D1, Barremian, Lower Cretaceous.

Type material: Holotype deposited in Museo de Ciencias Naturales y Antropológicas Juan Cornelio Moyano, Colección de Paleontología de Invertebrados, Mendoza, Argentina (MCNAM-PI) 24524.3; paratypes MCNAM-PI 24524.1, 24524.5, and 24524.6; Colección de Paleontología, Universidad de Buenos Aires, Ciudad Autónoma de Buenos Aires, Argentina (CPBA) 23706.1.

Remarks: Cataldo et al. (2019: 451) placed the species “not without hesitation” in Provalvatidae.

† ***Amplovalvatamansueta*** Pan, 1982 (Nom: 66)

Original source: Pan 1982: 431–432, pl. 2: figs 3, 4.

† ***Valvatamathiasi*** Esu & Girotti, 2018 (NEW)

Original source: Esu & Girotti, 2018: 49–54, figs 2, 3.

Type locality: Sambuca Lago Piccolo, Tavarnelle Val di Pesa, Tuscany, Italy.

Type horizon: Lower Pliocene.

Type material. Holotype Senckenberg Museum Frankfurt (SMF) 349126, from SLP3;

paratypes SMF 349127/3 and 349128/1, also Museo di Scienze della Terra of Sapienzia Universita (formerly Museo di Paleontologia), Roma, Italy, MPUR7-3959/50, 3960/80, 4153/20.

Remarks: the largest true *Valvata* known.

† “**Valvata (Cincinna) mengyinensis** (Grabau)” mentioned in Kobayashi (1983: 57) (NEW)

Remarks: probably an error for *Bithyniamengyinensis* Grabau, 1923, which is shown in the same figure as *Valvatasuturensis* by Grabau (1923: 161, fig. 7a–d (*Bithyniamengyinensis*), fig. 7e–g (*Valvatasuturalis*)).

***Valvatamicroscopica*** Nevill, 1889 (Nom: 68)

Type material: According to Ponder et al. (2014: 139) the holotype, originally deposited in Calcutta Museum, India, is apparently lost. Annandale and Kemp (1916: 347, text fig. 3) figured a “co-type” labelled as *Cyclostremamicroscopica*, which was reproduced by Ponder et al. (2014: 140, fig. 27).

Remarks: Ponder et al. (2014: 139ff) tentatively treated this species as *microscopica* (Nevill, 1877) (Truncatelloidea, Clenchiellidae).

**Cincinna (Sibirovalvata) sibirica middendorffi** Starobogatov & Zatravkin, 1985 (Nom: 69)

Remarks: Saito et al. (2018) provided molecular data from specimens from Nadezhdinsky District, Primorsky region, Russia.

† ***Valvataminima*** Hislop, 1859 (non Fuchs, 1877) (Nom: 69)

Type horizon: Upper Cretaceous (not Tertiary).

Type material: Lectotype designated and figured by Hartman et al. (2008: fig. 15A, B), reprinted by Bingle-Davis (2012: 132), deposited under PIMG 1251 (Palaeo Invertebrate Mesozoic Gastropod) at the Natural History Museum of the United Kingdom (NHMUK); the original Latin description was translated to English.

† “***Valvataminima*** Fuchs, 1877” (Nom: 69)

Remarks: As outlined this name is a junior homonym of *Valvataminima* Hislop, 1859. Accordingly, the name was replaced by *Pseudamnicolawelterschultesi* Neubauer, Harzhauser, Kroh, Georgopoulou & Mandic, 2014 (Hydrobiidae: Pseudamniocolinae). Neubauer et al. (2014b: 19) did not agree with Wenz (1928: 2439), who considered synonymy of *Valvataminima* Fuchs, 1877 with *Valvataserbica* Brusina, 1902 (an available name).

† ***Provalvataminor*** Cataldo, Lazo, Luci & Aguirre-Urreta, 2019 (NEW)

= *Provalvata* sp. in Lazo et al. (2017: 32, fig. 5k, l).

Original source: Cataldo et al. 2019: 451–453, figs 6 (1–8), 7 (1–8).

Type locality: Quebrada del Gastrópodo, Mendoza Province, Argentina.

Type horizon: La Tosca Member, Huitrín Formation, facies D1, Barremian, Lower Cretaceous.

Type material: Holotype deposited in Museo de Ciencias Naturales y Antropológicas Juan Cornelio Moyano, Colección de Paleontología de Invertebrados, Mendoza, Argentina (MCNAM-PI) 24523.3, paratypes MCNAM-PI 24523.2, 24523.4 and 24523.5; Colección de Paleontología, Universidad de Buenos Aires, Ciudad Autónoma de Buenos Aires, Argentina (CPBA) 23704.1.

Remarks: Cataldo et al. (2019: 451) placed the species “not without hesitation” in Provalvatidae.

† “***Costovalvataminuta***” mentioned in Youluo, 1978 (Nom: 70)

Original source: Youluo 1978 (not available from there, see above).

Type locality: Qingjiang Basin, Jiangxi, China.

Type horizon: Linjang Formation, Eocene.

Type material: Nanjing Institute of Paleontology and Geology, sample # 93414.

***Valvataminutissima*** Wattebled, 1884 (Nom: 70).

Type material: Ponder et al. (2014: fig. 2A–C) figured the holotype, which is stored in the Muséum national d´Histoire naturelle, Paris (MNHN-IM-2000-33594).

Remarks: Ponder et al. (2014: 126ff, figs 2, 4, 15, 33) confirmed synonymy with *Clenchiellapapuensis* Benthem Jutting, 1963 and provided details on morphology, SEM photographs of the operculum (fig. 4E, F), radula (fig. 5C, D), anatomy (fig. 15), and COI sequences (fig. 33) of this species, which is now treated as *Clenchiellaminutissima* (Wattebled, 1884) (Truncatelloidea, Clenchiellidae).

† ***Valvatamontanaensis*** Meek, 1876 (Nom: 71)

Remarks: In a published abstract of a conference, Canoy Illies and Hartmann (2018) designated a lectotype (USNM-PAL 2177a) and proposed (but not named) a new genus with this species as type species. Also, the type horizon is specified as the upper part of the Judith River Formation in Upper Missouri, Montana (Coal Ridge Member, Rogers et al. 2016).

***Valvatamontenegrina*** Glöer & Pešić, 2008 (Nom: 71)

Remarks: The holotype of *V.montenegrina* is figured by Glöer (2019: 203, fig. 254). Barcoding sequences of the COI gene are deposited by Falniowski et al. (2021) in GenBank with the numbers MZ027632 and MZ027633.

“***Valvatamontenegrinus*** Glöer & Pešić, 2008” in Glöer (2019: 203) (NEW)

Remarks: Misspelling of *Valvatamontenegrina* Glöer & Pešić, 2008 (no reason given for gender change).

† ***Valvatamulticarinata*** Hislop, 1859 (non Yen, 1946) (Nom: 73)

Type horizon: Upper Cretaceous (not Tertiary).

Type material: Lectotype designated and figured by Hartman et al. (2008: fig. 17A–D), reprinted by Bingle-Davis (2012: 132), stored under PIMG 1190 (Palaeo Invertebrate Mesozoic Gastropod) at the Natural History Museum of the United Kingdom (NHMUK); the original Latin description was translated to English.

***Valvatanowshahrensis*** Glöer & Pešić, 2012 (Nom: 75)

Holotype also figured by Glöer (2019: 213, fig. 267).

† “***Valvatanikosi***” mentioned in Esu and Girotti (2015: 78) (NEW)

Remarks: Mismatch of *Pyrgulanikosi* Esu & Girotti, 2015 and *Valvatapyramidula* Esu & Girotti, 2015, both described in the same paper.

***Cyclostomaobtusum*** Draparnaud, 1801 (Nom: 76)

Type material (10 syntypes, most of them juveniles or subadults) have been located by Vinarski and Kantor (2016: 269) in the Naturhistorisches Museum Wien (NHM), # 14704.

Remarks: Several Russian authors regard this species as valid, whereas most European authors consider this to be a synonym of *Valvatapiscinalis* O.F. Müller, 1774.

† ***Valvataoctonaria*** Brusina, 1902 (Nom: 76)

Remarks: The taxon was ranked as subspecies of *Valvatasimplex* Fuchs, 1870 (non Gould 1847) by Wenz (1928: 2476). According to Neubauer et al. (2014b: 20) its generic affiliation is currently uncertain and needs revision; the current status is *Muellerpaliaoctonaria* (Brusina, 1902) (Hydrobiidae).

† “***Valvataoecsensis*** Soós, 1934” mentioned in Papp (1953: 109), Schlickum (1978: 246, pl. 18: fig. 1), Stojaspal (1990: 651, pl. 1: fig. 2), and Harzhauser and Binder (2004: 10, pl. 3: figs 9–11).] (NEW)

Remarks: Neubauer et al. (2014b: 20) pointed out that the original name “*öcsensis*” in the cited publications was erroneously emended to “*oecsensis*”. However, the correct emendation following ICZN rules is “*ocsensis*”, since it is not derived from a German word (ICZN 32.5.2.1). Current status: *Valvataocsensis* Soós, 1934.

**Valvata (Pseudomegalovalvata) olkhonica** Bekman & Starobogatov, 1975 (Nom: 77)

Remarks: Sitnikova (2018: fig. 2C) figured the holotype.

**Valvatalewisivar.ontariensis** Baker, 1931 (Nom: 77)

Remarks: Recently, Hinchliffe et al. (2019) confirmed by DNA barcoding that, despite the open coiling of the shell, this taxon is genetically identical to and thus a junior synonym of *Valvatalewisi* Currier, 1868. The authors also provided good photographs of the shells of both taxa.

† “***Valvataoregonensis***” mentioned in Hanna (1922: 11) (Nom: 78)

Remarks: As already outlined by Henderson (1935: 190), this name has been introduced in express synonymy by Hanna (1922: 12: “This species was briefly described under two names [*V.whitei* and *V.calli*] in 1910 by Hannibal”). Since the two names previously published are both available, the name *Valvataoregonensis* is not valid.

“***Valvatacristatapalustris*** Kormos” twice mentioned in Motuz 1975: 57), also listed in the Global Names Index GNI (Nom. 79)

Remarks: As assumed in Haszprunar (2014) this is a confusion between *Valvatacristata* Müller, 1774 and *Lymnaeapalustris* Müller, 1774, both listed in Kormos (1912).

**Valvata (Cincinna) pamirensis** Starobogatov, 1972 (Nom: 79)

Type material: Type data were provided by Sitnikova et al. (2017: 259), who also figured the holotype (fig. 3G–I) (ZIN 1/241-1955) and paratype (ZIN 10/241-1955) from the type locality (# 10, fig. 3J–L) in the Zoological Institute of the Russian Academy of Sciences, St. Petersburg (ZIN).

† “***Valvatapanagilae***” [sic! not *panagile*] mentioned in Siodiropoúlou (2003: 41–42) (Nom: 79)

Remarks: This and four other fossil species (*V.catariane*, *V.olgae*, *V.mariae*, *V.theocleti*) from Pliocene – Pleistocene sediments of the Ptolemaida Basin (west Macedonia, Greece) are all extensively described and figured by Siodiropoúlou (2003). However, this PhD thesis never has been formally published, and thus the names are not available. I have also failed to detect any secondary reference using the name with a diagnosis or figure, which would make any of these names available.

**Valvata (Megalovalvata) lauta var. parvula** Kozhov, 1936 (Nom: 79)

Remarks: Sitnikova (2018: fig. 6D) illustrated the lectotype.

† ***Cincinnapenglaizhenensis*** Pan, 1982 (Nom: 81)

Original source: Pan 1982: 431, pl. 1: figs 22–23, pl. 2: figs 1, 2.

† “**Valvata (Atropidina) pileiformis**” mentioned in Youluo 1978 (Nom: 83)

Remarks: Not available from there (see above) or from Ye et al. (1996: 49; nomen nudum, no reference). I could not find a subsequent full reference on this species that would validate the name.

**Valvata (Liratina) baicalensis var. piligera** Lindholm, 1909 (Nom: 83)

Remarks: Sitnikova (1991: figs 1.1, 2.1–4,) figured spawn and embryos [as *Megalovalvatapiligerapiligera*]. Vinarski and Kantor (2016: 282) provided data on types, type locality with coordinates, distribution, and bionomics. Saito et al. (2018) added molecular data from specimens from Lake Baikal (Listvyanka), Oblast Irkutsk, Russia. Sitnikova (2018) illustrated the lectotype (fig. 6E) and specimens of morphs *nudicarinata* (fig. 6F) and *minor* (fig. 8D).

***Neritapiscinalis*** O.F. Müller, 1774 (Nom: 83)

Type material: Vinarski and Kantor (2016: 268) stated that they could not find type material in the Zoological Museum of Copenhagen as assumed by Kantor et al. (2011: 70).

† **Valvata (Valvata) platispira** Szőts, 1953 (NEW)

Original source: Szőts, 1953: 33, 145, pl. 2: figs 13–15.

Type locality: “Hosszúharasztos” (Harasztos quarry), Gánt, District Fejér, Hungary.

Type horizon: Eocene.

† **Valvata (Cincinna) polita** A.A. Ali-Zade, 1973 (NEW)

Original Source: A.A. Ali-Zade 1973: 155–156, pl. 45: figs 1a, 1b.

Type locality: Bozdag (hill), Lower Absheron Peninsula of Azerbaijan.

Type horizon: Lower Akchagylian, uppermost Pliocene.

Type material: Dr Pavel Frolov (pers. comm. via Dr. Paval Kijashko, 22 Feb 2022) provided information that the fossil molluscs described by A.A. Ali-Zade in 1973 are all stored in the Museum of Earth Sciences of Moscow State University (MES MSU). The holotype has inventory number (MES MSU # 28/308).

Remarks: judged from the figures, which show a non-circular aperture, this taxon is not a valvatid and is in need of revision.

† **Valvata (Cincinna) pontica** Pană, 1990 (NEW)

Original source: Pană, 1990: 63, pl. 1: figs 3–10.

Type locality: Valley Croitorului, village Sibiciul de Jos, district Buzău, Romania.

Type horizon: Upper Miocene, lower Pontium.

Type material: Holotype No. 674, Collection Laboratoire de Paléontologie III g, Bucarest, Romania.

† **Valvatasimplexvar.polycincta** Lörenthey, 1906 (Nom: 85)

Remarks: The name was synonymized with *V.simplexoctonaria* by Wenz (1928: 2476), a view shared by Neubauer et al. (2014b: 21). Since the parent species name is not available and has been replaced (see below under *Vatributarylvatasimplex*), the current status is *Muellerpaliahaszprunarioctonaria* Neubauer, Harzhauser, Kroh, Georgopoulou & Mandic, 2014 (Hydrobiidae).

**Valvata (Pseudovalvata) profundicola** Bekman & Starobogatov, 1975 (Nom: 87)

Remarks: Sitnikova (2018: fig. 12F) figured the reticulate pattern of the shell by SEM and provided photographs (fig. 2A) and measurements of paratypes.

† **Valvata (Borysthenia) pronaticina** Lindholm, 1932 (NEW)

Original source: Lindholm 1932: 17, pl. 3: fig. 8a–h.

Type locality: East border of creek Betekei, a tributary of river Ischim near Selim-Dzhevar; district Petropavlovsk, province Akmolinsk (now Astana), North Kazachstan.

Type horizon: Lower Pliocene.

Type material: stored in the Central Scientific Research Geological Exploration Museum named after F.N. Chernyshev (CNIGR) # 412–419/3355 (see Malchevskoya 1985: 211). 21 syntypes are deposited in the Zoological Institute of the Russian Academy of Sciences, St. Petersburg (ZIN 1/359-1935).

† “***Valvatacristatapslustris***” [sic] mentioned in Ye et al. (1996: 166) (NEW)

Remarks: probably misspelled and confused with *Stagnicolapalustris* (O.F. Müller, 1774).

“***Costovalvatapulchra***” mentioned in Ye et al. (1996: 47) (NEW)

Remarks: nomen nudum, not available.

***Neritapusilla*** Müller, 1774 (Nom: 88)

Original source: Müller 1774: 171 (# 357) referring to “Berl(in) Magaz(in) [Berlinisches Magazin: 4. B(and) p(agina) 268, t(abula) 8, f(igura) 26”, published 1769].

Type material: Not mentioned by Nekhaev et al. (2015). Vinarski and Kantor (2016: 265) failed to find type material in the Zoological Museum of Copenhagen.

Remarks, The figures (25 and 26)) in the article “Berlinisches Magazin” (an author is not identified) certainly refers to a species of Valvatidae and not to a neritid.

“***Valvatapygrncea***” mentioned at many webpages, e.g., https://commons.wikimedia.org/wiki/File:The_Quarterly_journal_of_the_Geological_Society_of_London_(1867)_(14595069379).jpg (NEW)

Remarks: Misspelling of *Valvatapygmaea* C.B. Adams, 1849 because of erroneous text recognition.

† ***Valvatapyramidula*** Esu & Girotti, 2015 (NEW)

Original source: Esu and Girotti 2015a: 76–78, figs 29–32.

Type locality: S-SW of Neos Erineos, Greece (GPS w66 = 38°16'45.22"N, 21°59'46.33"E).

Type horizon: Upper Lower Pleistocene, grey-yellow silty clays of the Synania Formation.

Type material in Senckenberg Museum Frankfurt (holotype SMF 345727, paratypes SMF 345728/3).

† “***Liratinaqikouensis***” mentioned in Youluo 1978 (Nom: 89)

Remarks: Not available from there (see above) or from Ye et al. (1996: 49; nomen nudum, no reference). I could not find a full reference on this species that would validate the name.

† **Valvata (Cincinna) racovetzae** Popova & Starobogatov (in Popova, Devyatkin & Starobogatov, 1970) (NEW)

Original source: Popova et al. 1970: 23 (pl. 1: fig. 3), 26.

Type locality: The Chuya Basin (or Chuya Steppe), southeastern part of the Altai Mountains, Russia.

Type horizon: Kyzylgir formation, Middle to Upper Pliocene.

Type material: Holotype deposited in the Zoological Institute of the Russian Academy of Sciences, St. Petersburg (ZIN 1/533-1968).

Remarks: Unfortunately this fossil species is not included in the review of freshwater gastropod taxa created by Starobogatov (Sitnikova et al. 2017).

† “**Valvata (Cincinna) rakovetzae**” mentioned in Popova et al. 1970: 26

Remarks: Probably a misspelling of † Valvata (Cincinna) racovetzae: (1) The figure legend at page 23 (first mention in the paper) states “*racovetzae*”. (2) The same paper includes the description of Anisus (Pseudocarinogyraulus) racovetzae (Hygrophila: Planorbidae; pp. 43, 44) and *Odhneripisidiumracovetzae* (Bivalvia: Pisidiidae; pp. 72, 74), showing the general preference of the authors for the latter spelling. (3) The second author, Bogachkin (1981: 33) listed a similar species as “Valvatacf.racovetzae Pop. Et Starob.”. On the other hand, Dr Pavel Kijashko (pers. comm. 22 Feb 2022) stated that “I believe that the name “*rakovetzae*” is a priority. It is indicated in the original description and on the original identification label by the hand of Starobogatov it is written: “*V.rakovetzae*, holotype”. Perhaps it is Starobogatov’s typo (it cannot be clarified now), but it does not contradict ICZN.” The matter may be decided by the Commission of Zoological Nomenclature.

† **Valvata (Cineinna) [sic!] *rehetaiensis*** Zhu G.-X., 1980 [non Youluo 1978] (Nom: 90)

Original source: Zhu 1980: 38, pl. 19: fig. 3, referred to Youluo (1978).

Remarks: Not available from Youluo 1978 (see above), but Zhu (1980) fulfils all requirements of validation (description and figure). The subgenus Cincinna is misspelled as *Cineinna*.

Type locality: Coastal region of Bohai, northeast China.

Type horizon: The lower part of the first section of the Eocene–Oligocene Shahejie Formation.

† “***Valvataringentis***” mentioned in Youluo 1978 (Nom: 91)

Remarks: Not available from there (see above). I could not find a subsequent full reference on this species, which would validate the name. The name “*A.* [*Amnicola*] *ringentis* Youluo” mentioned in Qu et al. (2006: 361) refers to another (likewise not available) name and species (Youluo 1978: 49, pl. 6: figs 29, 30, pl. 7: figs 19, 20).

† ***Valvatarobusta*** Martinson, 1882 (Nom: 91)

Original source: Martinson 1982: 70, pl. 16: figs 23, 24.

Type locality: Tsogt-Ovoo of Gobi desert, southeastern Mongolia.

Type horizon: Upper Cretaceous, Albian, Khukhtyk Formation.

Type material: Holotype No. 5577/4 deposited at Limnological Institute, Siberian Branch of the Russian Academy of Sciences. Paratypes: 10 in good condition, 8 in satisfactory condition.

† ***Valvatasayni*** Delafond & Deperet, 1893 (Nom: 93)

Remarks: Also listed as “ValvatasibinensisNeum.var.sayni Font.” by Jodot (1955: 601). As previously noted, Fontannes (1883: 440) only published a nomen nudum, which was later made available by Delafond and Depéret (1893: 47).

† ***Valvataserbica*** Brusina, 1902 (Nom. 94)

Remarks: As previously noted, Brusina’s name is a junior synonym of *Valvataminima* Fuchs, 1877, the latter is a junior homonym of *Valvataminima* Hislop, 1859. Accordingly, the Brusina/Fuchs name has been replaced by Neubauer et al. (2014b: 19) as *Pseudamnicolawelterschultesi* Neubauer, Harzhauser, Kroh, Georgopoulou & Mandic, 2014 (Hydrobiidae).

**Valvatacristatavar.sibirica** Middendorff, 1851 (Nom: 95)

Type material: Vinarski and Kantor (2016: 277) explained that Bogatov and Zatravkin (1992: 33) did not designate a lectotype as assumed by Prozorova and Starobogatov (1998: 56) but only listed a syntype (collected in Barnaul). This syntype is kept in Zoological Institute of the Academy of Sciences, St. Peterburg (ZIN), as # 1 under the name. Glöer (2019: 213, fig. 268: 5–7) also figured syntypes from the Naturalhistoriska Museet Goteborg (GNHM 4677).

Remarks: Andreeva et al. (2021: fig. 5A) published excellent photographs of specimens of the Taz River basin (western Siberia).

† “**Valvata (Valvata) simplex** Fuchs, 1870” (Nom: 95–96)

Remarks: As outlined in Haszprunar (2014: 95), the taxon is a junior homonym of Valvatatricarinatavar.simplex Gould, 1841 and is in fact a hydrobiid. Accordingly, the name has been replaced by Neubauer et al. (2014b: 19–20) with *Muellerpaliahaszprunari* Neubauer, Harzhauser, Kroh, Georgopoulou & Mandic, 2014 (Hydrobiidae). The same authors also renamed †*Valvataoctonaria* Brusina, 1902 (although only tentatively: “needs revision”) as *Muellerpaliahaszprunarioctonaria* (Brusina, 1902).

“***Valvataskniadica***” mentioned in Ye et al. (1996: 166) (NEW)

Remarks: Misspelling of Valvata (Aphanotylus) skhiadica Bukowski, 1895.

**Valvata (Cincinna) sorensis** Dybowski, 1886 (Nom: 97)

Type material: According to Sitnikova et al. (2015: 10) type material may be stored in the Collection of the Benedict Dybowski Zoological Museum, Ivan Franko National University, Lviv (Ukraine).

Remarks: Clewing et al. (2014: supplementary material) provided molecular data (as RU02/2). Sitnikova et al. (2015: 10–19, figs. 4C, D) considered Valvata (Cincinna) sorensis var. abbreviata Lindholm, 1909 as a junior synonym, provided photographs of syntypes and from specimens of several localities as well as an extensive and annotated citation record in the Russian literature in particular. Andreeva et al. (2021: fig. 3C) added excellent photographs from specimens of the Taz River basin (western Siberia).

***Valvataspirorbis*** Draparnaud, 1897 (Nom: 98)

Type material: According to Vinarski and Kantor (2016: 263) a single shell (syntype) is stored in the Naturhistorisches Museum Wien (NHM #14717).

† **Valvata (Cincinna) splendida** Szőts, 1953 (NEW)

Original source: Szőts, 1953: 33, 145–146, pl. 2: fig. 16.

Type locality: “Hosszúharasztos” (Harasztos quarry), Gánt, District Fejér, Hungary.

Type horizon: Upper Lutetian to lower Bartonian, Middle to upper Eocene.

† ***Valvatastevanovici*** Ilyina (in Stevanovich & Ilyina, 1982) (Iljina in Global Names Index GNI and Index of Organism Names ION) (Nom: 98)

Type material: Holotype stored in the Paleontological Institute, Russian Academy of Sciences (PIN 2220/587).

“***Valvataradiatulasubnaticina***” (NEW) mentioned at GBIF, Catalogue of Life, World Register of Marine Species, and at Mineralienatlas https://www.mineralienatlas.de/lexikon/index.php/FossilData?fossil=Valvata%20radiatula%20subnaticina.

Remarks: The name is a mistake based on the description of *Valvatasubnaticina* Łomnicki, 1886 (Nom: 100). There it is stated that fossils of forms similar to *Valvataradiatula* Sandberger, 1875 also occur at the type locality; the latter are clearly different from *V.subnaticina*, however.

“***Valvatacristata* monstr. *subscalaris***” mentioned in Baudon (1884: 294, pl. 9: fig. 19) (NEW)

Remarks: Obviously considered as a monstrosity, therefore not available.

† ***Liratinasubtilostriata*** Pan, 1980 (in Yü & Pan, 1980) (Nom: 102)

Original source: Yü and Pan 1980: 148, pl. 2: figs 7–10.

† “***Planorbissymmetricus*** Ludwig, 1865” mentioned in Haszprunar (2014: 103) (NEW)

Remarks: Misspelling for *Planorbissymmetrus* Ludwig, 1865; the current name is *Valvatasymmetra* (Ludwig, 1865).

† ***Valvatatanaiticus*** Sanko, 2007 (NEW)

Original source: Sanko 2007: 75–76, text fig. 52.

Type locality: Korotoyak section at the Upper Don river, District of Voronezh Oblast, Russia.

Type horizon: Deeper than Alexandrian Interglacial, Middle Pleistocene.

**Valvata (Pseudomegalovalvata) tenagobia** Bekman & Starobogatov, 1975 (Nom: 104)

Type material: According to Vinarski and Kantor (2016: 273) holotype and 15 paratypes in the Zoological Institute of the Russian Academy of Sciences, St. Petersburg (ZIN 1/122-1976 and ZIN 2/122-1976). The holotype is figured by Sitnikova (2018: fig. 2E).

“***Valvatatheotokii***” mentioned in Haszprunar (2014: 105) and Glöer and Hirschfelder (2019: 10) (NEW)

Remarks: Misspelling of *Valvatatheodokii* Locard, 1889.

“***Liratinatongbinzhenensis***” mentioned in Ye et al. (1996: 49, 50) (NEW)

Remarks: nomen nudum – not available.

† ***Valvatatransbaicalensis*** Martinson, 1956 [non 1961] (Nom: 105)

Original source: Martinson 1956: 21, text fig. 16; pl. 2: fig. 14.

Type locality: Mordoy area, east Sibiria (Transbaikalia), Russia.

Type horizon: Lower Cretaceous (Valanginian – Hauterivian).

***Cyclostomatricarinata*** Say, 1817 (Nom: 105)

Remarks: Yurco and Keeney (2018) provided microsatellite data to enable analyses of population genetics of this widespread species.

† ***Valvatatuostaiensis*** Wei, 1984 (Nom: 107)

Original source: Wei in Xinjiang Dizhi Ju 1984, 84, pl. 49: figs 3, 4.

Type locality: Wuyiju Tostai, Xinjang Province, China.

Type horizon: Taxihe Formation, Miocene.

† “***Liratinatuozhuangensis***” [not *Valvatatuozhuangensis* as stated] mentioned in Youluo 1978 (Nom: 107)

Remarks: Not available from there (see above), nor from Meyerhoff et al. (1991: 102), Ye et al. (1996: 47, 160, 285), Ryo et al. (2000: 11), or Lin et al. (2005: 56): all these citations are nomina nuda lacking descriptions, figures, or detailed reference. I could not find any full reference on this species that would validate the name.

† ***Valvataturbinata*** Stache, 1889 (NEW)

Original source: Stache 1889: 117, pl. 2: fig. 24.

Type locality: Caracaea-oogones, north of Cosina (today Hrpelje-Kozina), (5 km east of Trieste) Slovenia.

Type horizon: *Stomatopsis* horizon, Eocene.

Remarks: Because of the *Conus*-like shell, a very doubtful member of Valvatidae.

† ***Valvataturbinoides*** K.A. Ali-Zade, 1936 (NEW)

Original source: K.A. Ali-Zade 1936: 17, pl. 1: figs 28–30 (not seen, according to MolluscaBase (2022: see references).

Type locality: near Naftalan, west Azerbaijan.

Type horizon: Akchagylian, uppermost Pliocene and lower Pleistocene.

Remarks: Replacement name for *Valvataalta* K.A. Ali-Zade, 1932, a junior homonym of *Valvataalta* Deshayes, 1862 (now considered as *Bythinellaalta* (Deshayes, 1862), Bythinellidae). Not treated in the last 50 years.

“***Valvataturkmena***” mentioned in A.A. Ali-Zade 1967: 225) (NEW)

Remarks: Probably a misattribution of *Pyrgulaturkmena* A.A. Ali-Zade, 1967, described in the same volume.

“***Valvatapiscinalis*** (Müll.) var.uistopolitana Pop[ov]” (NEW) mentioned in Petrova and Linkina (2014: 113) (NEW)

Remarks: A misspelling of *Valvatacistopolitana* (see above).

“***Valvataumbilicata*** Parreyss” is also mentioned in Baudon (1884: 293) (Nom: 108)

† ***Valvataunicarinifera*** Hislop, 1859 (Nom: 108)

Type horizon: Upper Cretaceous (not Tertiary as stated by Hislop).

Type material: Lectotype designated and figured by Hartman et al. (2008: fig. 16A, B), reprinted by Bingle-Davis (2012: 132), deposited under PIMG 1239 (Palaeo Invertebrate Mesozoic Gastropod) at the Natural History Museum of the United Kingdom (NHMUK); the original Latin description was translated to English.

† “***Valvataunicariniferaunicarinifera***” mentioned in Bingle-Davis (2012: 104) (NEW)

Locality: near Kalmeshwar (station InL0096b), west of Nagpur, Savner Subdivision of Nagpur district in the state of Maharashtra, India.

Horizon: Cretaceous.

Material: “Holotype”: InS1159 (Appendix 2 (Nom: 137), fig. O; 1.5 mm × 1.7 mm) stored at University of North Dakota, Grand Forks, North Dakota, USA.

Remarks: Although freely available online, this PhD dissertation lacks ISBN or ISSN numbering, thus is not formally published. Moreover, in erecting a second subspecies of *Valvataunicarinifera* Hislop, 1859 by Bingle-Davis in the same work (see above for “*V.u.chiknaformis*”), the original taxon becomes the nominal subspecies and cannot be replaced. Thus, the specimens of Bingle-Davis are formally not named and remain to be described in accordance with the rules of ICZN.

**Valvatasimplexvar.unicincta** Lörenthey, 1906 (Nom: 109)

Remarks: As outlined above (see under *Valvatasimplex*) the parent species name is not available and has been replaced by Neubauer et al. (2014b: 21). The current status of this taxon, which needs revision, is *Muellerpaliaoctonariaunicincta* (Lörenthey, 1906) (Hydrobiidae).

†***Valvatauralica*** Popov, 1965 (NEW)

Original source: Popov 1965: 227, pl. 5: figs 25–29.

Type locality: Kama tributary of the Middle Volga, Russia.

Type horizon: Pleistocene, Sokol Suite of the Kinel Formation.

Type material: unknown.

Remarks: also mentioned by Danukalova and Morozova (2003: 80) and Matoshko et al. (2004: 21).

“***Valvatavaciani*** Nourn.” mentioned in Yahimovich et al. (2000: 65) (NEW)

Remarks: misspelling of *Valvatavanciana* Tournouër, 1875.

“***Valvatavenciana***” mentioned in Bogachev (1961: 74) (NEW)

Remarks: misspelling of *Valvatavanciana* Tournouër, 1875.

† **Cincinna (Cincinna) vinogradovskaense** [sic] Gozhik, 2002 (Nom: 110)

Type locality: near Vinogradovka village, Odesa oblast, Bolhrads’kyi district, Ukraine.

Type horizon: Miocene, Middle Pontian.

Type material: Holotype (coll. Gozhik, #3163) figured by Osipova et al. (2021: fig. 3D).

Remarks: The species name should be *vinog radovskaensis*, since both *Cincinna* and *Borysthenia* are feminine. “Cincinna (Cincinna) vinogradovkensis” mentioned in Haszprunar (2014: 110) is a misspelling. Currently considered a species of *Borysthenia* Lindholm, 1914.

**Valvata (Microcincinna) vystitiensis** Chernogorenko & Starobogatov, 1987 (Nom: 111)

Type material: Type data provided and holotype figured by Sitnikova et al. (2017: 257, fig. 3D–F).

† ***Valvataheidemariaewillmanni*** Neubauer, Harzhauser, Kroh, Georgopoulou & Mandic, 2014 (NEW).

Original source: Neubauer et al. 2014b: 22

Type locality: Vokasia-Tal, Kos, Greece.

Type horizon: Lower Pleistocene, Middle Iraki-Formation.

Type material: According to Neubauer et al. (2014b: 22) deposited in the Geological-Paleontological Institute, University of Kiel, no number provided.

Remarks: Replacement name for *Valvataheidemariaebicarinata* Willmann, 1981, a junior homonym of *Valvatabicarinata* Lea 1841.

† ***Valvatawindhauseni*** Parodiz, 1961 (Nom: 112)

Remarks: Parodiz (1969: 110) himself later assigned this species to the freshwater genus *Potamolithus* Pilsbry, 1896 (Truncatelloidea, Tateidae).

† ***Valvatayongkangensis*** Yü, 1980 (Nom: 112)

Original source: Yü and Pan 1980: 147–148, pl. 2: figs 3, 4.

Type horizon: Middle Jurassic.

† ***Valvatazhongjiangensis*** Pan, 1982 (Nom: 113)

Original source: Pan 1982: 430, pl. 1: figs 18–21.

† “***Valvatazhouqingzhuangensis***” mentioned in Youluo 1978 (Nom: 113)

Remarks: Not available from there (see above) or from Ye et al. (1996: 48; nomen nudum, no reference). I could not find any full reference on this species that would validate the name.
